# The relationship of adverse childhood experiences of pregnant women with healthy lifestyle behaviours and birth attitudes: A cross-sectional study

**DOI:** 10.1371/journal.pone.0341601

**Published:** 2026-02-05

**Authors:** Esra Cevik, Pelin Palas Karaca

**Affiliations:** Faculty of Health Sciences, Department of Midwifery, Assistant Professor, Balikesir University, Balikesir, Turkey; PLOS ONE, UNITED KINGDOM OF GREAT BRITAIN AND NORTHERN IRELAND

## Abstract

**Introduction:**

This study was conducted to determine the relationship between negative childhood experiences of pregnant women and health-promoting lifestyle behaviours and childbirth attitudes.

**Methods:**

The cross-sectional study was conducted with 468 pregnant women between March 2023 and January 2024. Data were collected using a descriptive characteristics form, an adverse childhood experiences questionnaire (ACEQ), a health-promoting lifestyle profile (HPLP-II), and a childbirth attitude questionnaire (CAQ). The study reporting followed the STROBE checklist.

**Results:**

Participants’ mean scores were as follows: ACEQ 1.10 ± 1.84, CAQ 39.91 ± 12.18, and HPLP-II 138.64 ± 23.71. Linear regression analysis showed that HPLP-II scores were significantly lower in non-employed women (*β* = −0.194; 95% CI: −14.06, −5.44; *p* = 0.000) and smokers (*β* = −0.104; 95% CI: −12.05, −1.05; *p* = 0.020). Lower income perception was significantly associated with decreased HPLP-II scores (*β* = −0.170; 95% CI: −11.34 to −3.48; *p* = 0.000). Additionally, higher ACEQ scores were also significantly related to lower HPLP-II scores (β = −0.092; 95% CI: −2.29 to −0.06; *p* = 0.037). Regarding CAQ, scores were significantly higher among participants with lower income perception (*β* = −0.200; 95% CI: −6.35, −2.59; *p* = 0.000), those who consumed alcohol during pregnancy (*β* = −0.322; 95% CI: −16.36, −9.70; *p* = 0.000), and those with higher ACEQ scores (*β* = 0.158; 95% CI: 1.09, 1.58; *p* = 0.000).

**Discussion:**

Pregnant women’s negative childhood experiences affect both birth anxiety and health-promoting lifestyle behaviours.

## Introduction

Pregnancy is a period characterized by physical, hormonal, psychological, and social changes, requiring women to adapt to it [1]. Maintaining health-promoting lifestyle behaviours during this process is critical for this adaptation [2]. Health-promoting lifestyle behaviours support individual health, enhance well-being, and improve perceived control over health [3]. Health-promoting behaviour refers to the actions and decisions through which individuals actively engage in maintaining or improving their well-being, aiming to achieve an optimal balance of physical, psychological, and social health. These behaviours include regular physical activity, smoking and alcohol avoidance, balanced nutrition, weight management, effective communication, stress control, and psychological well-being. Pregnant women who adopt these behaviours experience fewer complications during prenatal, intrapartum, and postpartum periods [3,4]. In the long term, such habits positively affect both maternal and child quality of life [5]. Recent studies suggest that adverse childhood experiences (ACEs) may negatively influence health-promoting lifestyle behaviours [6]. In this study, health-promoting lifestyle behaviours were evaluated using the Health-Promoting Lifestyle Profile II (HPLP-II), which includes six subdimensions: nutrition, physical activity, stress management, health responsibility, interpersonal relations, and spiritual growth. The subdimension health responsibility refers to an individual’s active sense of accountability for their own well-being. It includes maintaining personal health, being informed about health issues, and seeking professional help when necessary. Physical activity involves engaging in regular exercise at light, moderate, or vigorous levels as part of a planned lifestyle routine to maintain fitness and health. Nutrition implies maintaining a balanced and adequate diet through the conscious selection of foods that support physical and psychological well-being. Spiritual growth refers to the individual’s conscious effort to find meaning in life, recognize inner potential, and work toward personal goals. İnterpersonal relations describe the individual’s ability to establish meaningful, supportive, and trusting relationships, and to express emotions and thoughts effectively. Stress management reflects the ability to identify sources of tension and use effective cognitive and behavioural strategies to reduce or cope with stress [7]. Considering all these dimensions together, assessing modifiable lifestyle factors that women can control during pregnancy and providing appropriate interventions when necessary are crucial for both maternal and fetal health.

ACEs or childhood adversities include experiences of stress, neglect, abuse (e.g., physical, sexual, emotional), household dysfunctions (such as a parent who abuses substances, a parent with mental health problems), or potentially traumatic events children experience before the age of 18 [8,9]. The World Health Organization (WHO) refers to ACEs as the most intense and frequent source of stress children may experience early in life and addresses them under the headings of neglect, violence between parents or caregivers, other serious household dysfunctions (such as alcohol and substance abuse), and violence. Individuals with this experience are at increased risk of physical and mental health problems, risky health behaviours, impaired quality of life, smoking, alcohol intake, chronic health problems, obesity, sleep problems and early death in adulthood [8,10], learning difficulties, attention and behavioural problems in childhood [11]. Individuals with ACEs may start sexual intercourse earlier, have a higher number of sexual partners, have adolescent pregnancies, and be more likely to contract sexually transmitted infections [12]. It is emphasized that the lifetime rate of girls aged 0–17 years exposed to ACEs is 26.1% and this rate is similar globally [13].

There is increasing evidence linking ACEs with maternal morbidity and mortality. ACEs are associated with pregnancy complications, preterm birth, low birth weight, NICU admissions, and developmental problems in infants [1,8]. They can also lead to heightened anxiety during pregnancy, role confusion in motherhood, and impaired bonding with the infant [9]. This may adversely influence women’s birth attitudes. Birth attitude reflects a woman’s behaviours, perceptions, and stance toward childbirth. Research indicates that pregnant women exposed to emotional abuse, neglect, or cumulative childhood trauma report greater fear of childbirth [14], which has been linked to anxiety, poor coping, elective Caesarean delivery, postpartum depression, and both physical and psychological difficulties [15,16].

Along with the direct health consequences, ACEs have been linked to unintended pregnancy, prenatal depression, smoking, intimate partner violence, and perinatal mental health disorders [12,17]. These findings highlight the need to screen expectant mothers for ACEs during pregnancy to identify existing health risks early, implement preventive strategies, and promote maternal and child well-being [18,19]. Despite growing awareness of the impact of early-life adversity on adult health, studies specifically examining ACEs in the context of pregnancy remain limited [12,18,20]. Moreover, recent literature increasingly underscores childbirth as a potentially traumatic experience, particularly among vulnerable populations, with emphasis on its association with posttraumatic stress symptoms and negative emotional outcomes [21]. Identifying the factors that increase the risk of traumatic birth is therefore critical. In this context, examining health-promoting lifestyle behaviours alongside the influence of ACEs may address a significant gap in the literature.

This study is based on life course theory, which suggests that early life exposures and experiences, including adversities, accumulate and influence lifelong health trajectories and shape behaviours, attitudes, and outcomes in adulthood [22]. Using this framework, our study aims to explore the ways in which adverse childhood experiences (ACEs) may shape pregnant women’s health-promoting lifestyle behaviours and attitudes towards childbirth.

## Method

### Study design

The cross-sectional study was conducted in the obstetrics outpatient clinic between March 2023 and January 2024.

### Study setting and sampling

The study population consisted of pregnant women who applied to the gynaecology outpatient clinic of Balikesir University Health Practice and Research Hospital. This cross-sectional study adhered to the Strengthening the Reporting of Observational Studies in Epidemiology (STROBE) guidelines for cross-sectional studies, with detailed adherence outlined in the “Checklist of items that should be included in the cross-sectional studies” [23].

The sample size in the study was aimed to reach at least 384 people at 50% unknown prevalence, 5% deviation, and 95% confidence level, and the study was finalised with 468 people who met the acceptance criteria. Participants were recruited using a convenience sampling method. Women who were 18 years of age or older, Turkish-speaking and without language or communication barriers, not diagnosed with high-risk pregnancy (e.g., gestational diabetes, preeclampsia), who were over 28 weeks of gestation, and agreed to participate in the study were included. The dependent variables were health-promoting lifestyle behaviours and birth attitude.

The independent variables were childhood adverse experiences, age, educational status, employment status, income perception, family type, planned last pregnancy, smoking during pregnancy, alcohol use during pregnancy, gestational week, number of pregnancies, number of curettages, number of miscarriages. Given the sensitivity of the subject and the vulnerability of the target group, special attention was paid to ethical principles in the study. The purpose and content of the study were clearly explained to the participants and both written and verbal consents were obtained. Data were collected by the researcher in an environment providing confidentiality of the participants and where they could be alone. The ACE Questionnaire (ACEQ) was completed by the participants on their own and voluntarily to prevent possible guided responses. Participants were informed that they could withdraw from the study at any time without any justification.

### Instruments

The data of the study were collected through face-to-face interviews with pregnant women in pregnancy outpatient clinics of Balikesir University Health Practice and Research Hospital. Data were collected using the Descriptive Characteristics Form, Adverse Childhood Experience Questionnaire (ACEQ), Health-Promoting Lifestyle Profile II (HPLP II), and the Childbirth Attitudes Questionnaire (CAQ).

#### Descriptive characteristics form.

The Descriptive Characteristics Form, which was prepared in line with the literature, consisted of questions addressing the sociodemographic (age, family type, income status, alcohol, smoking) and obstetric (number of pregnancies, number of miscarriages, number of curettages) characteristics.

#### Adverse Childhood Experience Questionnaire (ACEQ).

The Centers for Disease Control and Kaiser Permanente developed the Adverse Childhood Experiences (ACEs) scale to find negative experiences in childhood [24,25]. A Turkish validity and reliability study was conducted by Gunduz et al. [26], consisting of 10 items about childhood traumas. The Turkish form of the scale consists of a single factor. There is only the ‘yes’ option for the questions, and otherwise, they are left blank. The ACEQ total score ranges between 0 and 10. There is no cutoff value. In the Turkish adaptation of the scale, Cronbach’s alpha value was found to be 0.740 and 0.810 in our study.

#### Health-Promoting Lifestyle Profile II (HPLP-II).

The scale was originally developed by Walker et al. in 1996 [27] and later adapted into Turkish by Bahar et al. in 2008. The Health-Promoting Lifestyle Profile II (*HPLP-II*) evaluates six distinct dimensions of health-promoting behaviours. These dimensions are: Health Responsibility, which involves monitoring personal health, seeking appropriate medical assistance, and being accountable for one’s well-being; Physical Activity, which includes engaging in regular exercise of varying intensity as part of a planned lifestyle routine; Nutrition, reflecting balanced, conscious, and consistent dietary choices; Spiritual Growth, which captures personal development through inner peace, self-awareness, and pursuit of meaning in life; Interpersonal Relations, involving the establishment and maintenance of supportive and communicative relationships; and Stress Management, which entails recognizing sources of stress and using behavioural or cognitive strategies to manage tension. Each subscale represents a behavioural domain that contributes to maintaining or improving physical and psychological health. Each item is rated on a 4-point Likert scale ranging from 1 (never) to 4 (routinely). The total score is calculated by adding all item responses, with possible scores ranging from 52 (lowest) to 208 (highest). As the score obtained from the scale increases, it shows that individuals develop positive health-promoting lifestyle behaviours. In the Turkish adaptation of the scale, Cronbach’s alpha value was found to be 0.920 [28] and 0.941 in our study.

#### Childbirth Attitudes Questionnaire (CAQ).

The scale was developed by Lowe in 2000 [29], consisting of 16 items evaluating labour anxiety and adapted into Turkish by Dönmez et al. in 2014. It is a four-point Likert-type scale, and its total score consists of the average of the answers given, with the lowest score being 16 and the highest 64. A high scale total score indicates high labour anxiety. Cronbach’s alpha value was 0.920 in the Turkish adaptation of the scale [30] and 0.942 in our study.

### Data collection

The study was initiated after obtainment of all permissions required (ethics committee approval, institutional approval, and verbal and written informed consent from the participants). Before the data collection phase, a pilot study of the forms and scales to be used in the study was conducted with 10 participants to assess the study applicability and the questions comprehensibility. In the pilot study phase, the 10 participants were selected in accordance with the same inclusion and exclusion criteria applied to the main sample. To obtain feedback on the comprehensibility and feasibility of the forms and scales used, participants were asked open-ended questions such as “Are there any items that you find unclear, complicated, or requiring further explanation?” and “What are your views on the overall applicability and content of the form?” This evaluation was conducted through face-to-face interviews by the researchers. Since no negative feedback regarding the clarity of the questions or the research methodology was received, no changes were made to the forms and scales. These pilot study procedures were conducted meticulously to support the study reliability and validity.

### Ethical considerations

The rules of the Declaration of Helsinki were followed in this study. Permission was obtained from the ethics committee of Balikesir University Health Sciences Non-Interventional Research (Date: 06.12.2022, Number: 2022/99), from the chief physician of Balikesir University Health Practice and Research Hospital (Date: 06.01.2023, Number: 214402), and written and verbal consent from the participants.

### Statistical analysis

Data were analysed using SPSS version 25. Descriptive statistics was presented as numbers, percentages, means, and standard deviations. Before the analyses, the normality of the distribution of continuous variables was assessed using the Shapiro–Wilk test and by evaluating skewness and kurtosis coefficients, which were within the acceptable range of ±1.5. For comparison of two independent groups, the independent samples t-test was used. For comparisons involving three or more groups, One-Way ANOVA followed by Tukey HSD post-hoc test was applied. These tests were chosen based on the assumption of normally distributed continuous outcomes and homogeneity of variances. Pearson correlation was used to assess linear relationships between continuous variables. To identify independent predictors of health-promoting lifestyle behaviours and birth attitudes, multiple linear regression analyses were conducted using the Backward method. Variables statistically significant in univariate analyses or supported by existing literature were included in the model to control for potential confounders. All major assumptions of linear regression—including linearity, normality of residuals, homoscedasticity, independence of errors (Durbin-Watson), and multicollinearity (*VIF* < 5) were checked and met prior to the analysis.

## Results

Among the pregnant women (*n* = 468), 41.2% were high school graduates, 66.5% were not working, 85.0% had nuclear family structure, 72.2% had planned pregnancy, 16.9% smoked, and 10% used alcohol. Regarding perceived income, when asked “How do you perceive your income level?”, 62.8% of the participants answered that they considered their income level to be “middle” (Table 1).

**Table 1:** Distribution of pregnant women’s sociodemographic and obstetric characteristics (*n* = 468).

Variable	*n*	%
**Educational status**		
Primary school	27	5.8
Secondary school	71	15.2
High school	193	41.2
University and above	177	37.8
**Employment status**		
Employed	157	33.5
Unemployed	311	66.5
**Perceived income level**		
Income less than expenses (Low)	22	4.7
Income equal to expenses (Middle)	294	62.8
Income greater than expenses (High)	152	32.5
**Type of family**		
Nucleus	398	85.0
Extended	40	8.5
Broken family	30	6.5
**Planned pregnancy**		
Planned	338	72.2
Unplanned	130	27.8
**Smoking during pregnancy**		
Yes	79	16.9
No	389	83.1
**Alcohol use during pregnancy**		
Yes	47	10.4
No	421	89.6
**Total**	**468**	**100.0**

The pregnant women’s mean age was 28.51 ± 4.75; the mean gestational week, 33.35 ± 3.71; the mean number of pregnancies, 1.76 ± 1.01; the mean number of curettages, 0.11 ± 0.39; the mean number of miscarriages, 0.17 ± 0.49; the mean ACEQ score, 1.10 ± 1.84; the mean CAQ score, 39.91 ± 12.18; and the mean HPLP-II score, 138.64 ± 23.71. The HPLP-II subdimension mean scores were 26.13 ± 4.83 for Spiritual Development, 25.71 ± 4.91 for Interpersonal Relationships, 23.66 ± 4.38 for Nutrition, 17.75 ± 5.33 for Physical Activity, 24.37 ± 4.78 for Health Responsibility, and 21.00 ± 4.38 for Stress Management. ACE exposure was common among expectant mothers, with almost half (42.3%) reporting at least one ACE before the age of 18 years, 15.4% reporting one ACE, 11.5% reporting two ACEs, and 15.4% reporting three or more ACEs, not shown in the table (Table 2).

**Table 2:** Distribution of the research group according to continuous variables (*n* = 468).

Variable	Mean *±* SD	Min-Max
**Age**	28.51 ± 4.75	21-42
**Pregnancy week**	33.35 ± 3.71	28-40
**Number of pregnancies**	1.76 ± 1.01	1-8
**Number of curettages**	0.11 ± 0.39	0-4
**Number of miscarriages**	0.17 ± 0.49	0-4
**ACEQ**	1.10 ± 1.84	0-10
**CAQ**	39.91 ± 12.18	16-64
**HPLP-II**	138.64 ± 23.71	52-208
Spiritual Development	26.13 ± 4.83	9-36
Interpersonal Relationships	25.71 ± 4.91	9-36
Nutrition	23.66 ± 4.38	9-36
Physical Activity	17.75 ± 5.33	8-32
Health Responsibility	24.37 ± 4.78	9-36
Stress Management	21.00 ± 4.38	8-32

SD: Standard deviation.

In the study group, HPLP-II score was significantly higher in those who had a university degree and higher education (*F* = 7.525, *p* = 0.001), were employed (*t* = 4.866, *p* = 0.001), had a good income perception (*F* = 19.116, *p* = 0.001), whose last pregnancy was planned (*t* = 3.093, *p* = 0.001), who did not smoke (*t* = 2.672, *p* = 0.009) nor drink alcohol (*t* = 2.686; *p* = 0.001) during pregnancy. HPLP-II score did not differ significantly according to family type (*p* > 0.05).

High CAQ scores of pregnant women indicate high anxiety, and labour anxiety is significantly higher in primary school graduates (*F* = 3.746, *p* = 0.011), in those with poor income perception (*F* = 19.331, *p* = 0.001), those whose last pregnancy was not planned (*t* = 3.125, *p* = 0.002), and those who used alcohol during pregnancy (*t* = 6.397; *p* = 0.001). CAQ score does not show statistically significant difference according to family type and smoking status during pregnancy (*p* > 0.05) (Table 3).

**Table 3:** Evaluation of HPLP-II and CAQ scores according to sociodemographic characteristics (*n* = 468).

Variable	*n*	HPLP-II Mean *± SD*	CAQ Mean *± SD*
**Educational status**			
Primary school^a^	27	125.25 ± 20.12	46.40 ± 14.19
Secondary school^b^	71	133.40 ± 25.69	41.38 ± 11.06
High school^c^	193	137.50 ± 23.21	39.73 ± 12.31
University and above^d^	177	144.03 ± 22.63	38.52 ± 11.85
p* **Post-hoc**		0.001 a = b = c < d	0.011 a > b = c = d
**Employment status**			
Employed	157	145.98 ± 24.04	39.99 ± 11.84
Unemployed	311	134.94 ± 22.69	39.87 ± 12.36
p**		0.001	0.918
**Perceived income level**			
Low^a^	22	114.54 ± 26.69	49.95 ± 12.34
Middle^b^	294	137.08 ± 23.23	41.27 ± 12.43
High^c^	152	145.16 ± 21.45	35.81 ± 10.11
p* **Post-hoc**		0.001 a < b < c	0.001 a > b > c
**Type of family**			
Nucleus^a^	398	138.78 ± 22.67	39.70 ± 12.21
Extended^b^	40	136.85 ± 29.82	42.10 ± 11.16
Broken family^c^	30	139.30 ± 28.52	39.76 ± 13.05
p*		0.876	0.495
**Planned pregnancy**			
Planned	338	140.93 ± 21.90	38.83 ± 11.68
Unplanned	130	132.71 ± 27.07	42.72 ± 13.02
p**		0.001	0.002
**Smoking use during pregnancy**			
Yes	79	131.30 ± 27.57	41.59 ± 13.11
No	389	140.14 ± 22.60	39.57 ± 11.97
p**		0.009	0.178
**Alcohol use during pregnancy**			
Yes	47	129.89 ± 26.04	52.68 ± 14.76
No	421	139.62 ± 23.27	38.48 ± 10.99
p**		0.007	0.001

SD: Standard deviation; *F: ANOVA test (Post hoc: Tukey HSD); **, Student’s t test; HPLP-II: Health-Promoting Lifestyle Profile II; CAQ: Childbirth Attitudes Questionnaire.

When the relationship between continuous variables and the HPLP-II Scale in the research group is examined, CAQ score (*r* = −0.146, *p* = 0.002) and ACEQ score (*r* = −0.136, *p* = 0.003) decrease, while HPLP-II score increases. When the correlation between HPLP-II and its subdimensions is examined, the HPLP-II score increases as the Spiritual Development (*r* = 0.856, *p* = 0.000), Interpersonal Development (*r* = 0.849, *p* = 0.000), Nutrition (*r* = 0.804, *p* = 0.000), Physical Activity (*r* = 0.711, *p =* 0.000), Health Responsibility (*r* = 0.888, *p* = 0.000), and Stress Management scores increase. There was no statistically significant relationship between HPLP-II score and age, gestational week, number of pregnancies, number of curettage, and number of abortions (*p* > 0.05) (Table 4).

**Table 4:** Association of Continuous Variables and ACEQ Score with HPLP-II and CAQ Scores (*n* = 468).

Variable	HLBS-II		CAQ	
	*r*	*p*	*r*	*p*
**CAQ**	−0.146	0.002	1	
**HLBS-II**	1		−0.146	0.002
Spiritual Development	0.856	0.000	−0.099	0.032
Interpersonal Development	0.849	0.000	−0.081	0.080
Nutrition	0.804	0.000	−0.047	0.306
Physical Activity	0.711	0.000	−0.234	0.000
Health Responsibility	0.888	0.000	−0.093	0.044
Stress Management	0.874	0.000	−0.156	0.000
**Age**	−0.035	0.448	−0.026	0.572
**Pregnancy week**	−0.045	0.330	−0.033	0.480
**Number of pregnancies**	−0.067	0.146	0.019	0.678
**Number of curettages**	0.040	0.390	−0.024	0.604
**Number of miscarriages**	−0.070	0.129	−0.029	0.534
**ACEQ**	−0.136	0.003	0.202	0.000

CAQ: Childbirth Attitudes Questionnaire; ACEQ: Adverse Childhood Experience Questionnaire; HPLP-II: Health-Promoting Lifestyle Profile II.

When the relationship between continuous variables and CAQ was examined in the research group, as the HPLP-II score decreased (*r* = −0.146, *p* = 0.002) and the ACEQ score increased (*r* = 0.202, *p* = 0.000), labour anxiety increased. When the correlation between CAQ and HPLP-II and their subdimensions are examined, the participants’ labour anxiety increases as the Spiritual Development (*r* = −0.099, *p* = 0.032), Physical Activity (*r* = −0.234, *p* = 0.000), Health Responsibility (*r* = −0.093, *p* = 0.044) and Stress Management scores decrease. There was no statistically significant relationship between CAQ and age, gestational week, number of pregnancies, number of curettages, number of abortions, Interpersonal Development and Nutrition scores (*p* > 0.05) (Table 4).

Multiple linear regression analysis using the backward elimination method was conducted by including variables statistically significant in univariate analyses. The final model explained 12.4% of the variance in HPLP-II scores (*R²* = 0.368, Adjusted *R²* = 0.124, *F* = 12.027, *p* = 0.000). Not working was associated with significantly lower HPLP-II scores (*β* = –0.194; *B* = –9.754; 95% CI: –14.06 to –5.44; *p* = 0.000), indicating that participants who were unemployed engaged in fewer health-promoting behaviours during pregnancy. Similarly, smoking during pregnancy was negatively associated with HPLP-II scores (*β* = –0.104; *B* = –6.553; 95% CI: –12.05 to –1.05; *p* = 0.020), suggesting reduced engagement in behaviours such as healthy eating, exercise, and stress management among smokers. Perceived income level showed a positive association with HPLP-II scores (*β* = 0.170; *B* = 7.411; 95% CI: 3.48 to 11.34; *p* = 0.000), indicating that participants who reported higher income perception also reported more health-promoting behaviours. Additionally, a higher ACEQ score, reflecting greater exposure to adverse childhood experiences, was significantly associated with lower HPLP-II scores (*β* = –0.092; *B* = –1.184; 95% CI: –2.29 to –0.06; *p* = 0.037). This suggests that early life adversity may negatively impact engagement in health-promoting behaviours during pregnancy. Variables such as educational level, planned pregnancy, alcohol use during pregnancy, and CAQ scores, although initially significant in univariate analysis, were excluded from the final model (*p* > 0.05). The regression model for CAQ scores explained 19.4% of the variance (*R²* = 0.199, Adjusted *R²* = 0.194, *F* = 38.486, *p* = 0.000). Alcohol use during pregnancy was the strongest predictor of higher CAQ scores (*β* = –0.322; *B* = –13.035; 95% CI: –16.36 to –9.70; *p* = 0.000), indicating that participants who consumed alcohol during pregnancy were more likely to experience fear, anxiety, or negative attitudes toward childbirth. Lower perceived income level was also significantly associated with higher CAQ scores (*β* = –0.200; *B* = –4.472; 95% CI: –6.35 to –2.59; *p* = 0.000), suggesting that socioeconomic disadvantage may contribute to negative childbirth attitudes. Finally, an increase in ACEQ score was positively associated with CAQ scores (*β* = 0.158; *B* = 1.040; 95% CI: 1.09 to 1.58; *p* = 0.000), indicating that participants with higher levels of childhood adversity reported greater fear or negative expectations about childbirth. Educational level, planned pregnancy status, and HPLP-II scores, though initially significant, were not retained in the final CAQ model (*p* > 0.05) (Table 5).

**Table 5:** Evaluation of independent variables and HPLP-II and CAQ scale scores according to linear regression analysis (*n* = 468).

HPLP-II	*B*	SE	*ß*	*p*	%95 C.I.	
					Lower	Higher
**Employment status**	−9.754	2.191	−0.194	0.000	−14.06	−5.44
**Perceived income level**	7.411	2.000	0.170	0.000	3.48	11.34
**Planned pregnancy status**	−4.454	2.374	−0.084	0.061	−9.11	.21
**Smoking during pregnancy**	−6.553	2.797	−0.104	0.020	−12.05	−1.05
**Alcohol use during pregnancy**	−6.136	3.486	−0.078	0.079	−0.71	12.98
**ACEQ**	−1.184	0.567	−0.092	0.037	−2.29	−0.06
**CAQ**	***B***	**SE**	**ß**	***p***	**%95 C.I.**	
					**Lower**	**Lower**
**Perceived income level**	−4.472	0.958	−0.200	0.000	−6.35	−2.59
**Alcohol use during pregnancy**	−13.035	1.697	−0.322	0.000	−16.36	−9.70
**ACEQ**	1.040	0.279	0.158	0.000	1.09	1.58

*B:* Regression coefficient; *ß:* Standardized ß; *SE:* Standard error; *CI:* Confidence interval;

*R* = 0.368, *Adjusted R*^*2*^ *= 0*.124, *F =* 12.027, *p =* 0.000.

*B:* Regression coefficient; *ß:* Standardized ß; *SE:* Standard error; *CI:* Confidence interval;

*R =* 0.199, *Adjusted R*^*2*^ = 0.194, *F* = 38.486, *p* = 0.000.

HPLP-II Variables included in the model: Educational status: (primary school:1, secondary school:2, high school:3, university and above:4), employment status: (working:1, not working:2), income perception: (poor:1, medium:2, good:3), planned pregnancy: (planned:1, not planned:2), smoking during pregnancy: (yes:1, no:2), alcohol use during pregnancy: (yes:1, no:2), Birth Attitude Scale (continuous), Childhood Adverse Experiences Scale: (continuous).

CAQ Variables included in the model: Educational status: (primary school:1, secondary school:2, high school:3, university and above:4), income perception: (poor:1, moderate:2, good:3), planned pregnancy: (planned:1, not planned:2), alcohol use during pregnancy: (yes:1, no:2), HPLP-II Scale (continuous), ACEQ: (continuous).

## Discussion

Adverse childhood experiences are a major public health problem [8,13]. The overall prevalence of at least one ACE ranged from 20.1% to 82% [9,10,12,17,31–33]. Approximately 5 out of 10 pregnant women (42.3%) who participated in our study stated that they had at least one adverse childhood experience in the first 18 years of their lives. ACE exposure is common among expectant mothers and 15.4% of those who reported ACEs reported one ACE, 11.5% reported two, and 15.4% reported three or more. Given the high prevalence of ACEs, individuals who experience them need interventions to reduce potential adverse health outcomes later in life. These interventions are important not only in terms of alleviating existing symptoms, but also in terms of the effects of early experiences that remain throughout an individual’s life. As a matter of fact, according to the theory of lifelong development put forward by Halfon and Hochstein (2002), early life exposures such as to ACEs accumulate over time and create lasting effects on both physical and mental health, shaping the individual’s behaviours, attitudes, and health-related choices in adulthood [22]. In our study, the mean ACEQ score of pregnant women was 1.10 ± 1.84. It was observed that the mean ACEQ score was like other studies conducted with pregnant women [8,12,18,20,34].

ACEs are associated with early physical morbidity and mortality, with people exposed to 6 or more ACEs dying 20 years earlier on average. Adverse childhood experiences contribute to adverse pregnancy outcomes [8,13], poor postnatal mental health, and impaired or delayed attachment, with mental health sequelae [13], low quality of life potential, and early death [10]. High ACE scores were associated with a 2-fold increased risk of hypertensive disorders and a 1.13-fold increase in preterm birth per additional ACE point [8,13]. It is therefore an issue requiring urgent intervention and a solution. Unfortunately, it is not included in screening programs in many countries. At this point, it is emphasized that screening expectant mothers for ACEs is essential to identify those at risk and to guide appropriate psychosocial interventions aimed at improving maternal and fetal health outcomes [8,18,19]. Training health professionals, psychiatrists, and counsellors in early intervention for children exposed to adverse experiences are effective measures to minimize the impact of adverse childhood experiences in later life [35]. Although the ACEQ captures experiences before age 18, the broad participant age range (21–42 years) may have introduced variability in health behaviours and birth attitudes. While age was not a statistically significant predictor in our regression models, future research should examine whether age influences how ACEs are internalized and expressed during pregnancy.

Expectations and experiences related to childbirth have a multifaceted structure in which women experience both positive and negative emotions together. In this process, they may experience positive emotions such as joy and hope as well as negative emotions such as anxiety, worry, and fear [36]. Especially women who have had negative experiences in childhood may experience such fears more intensely. In this study, it was observed that labour anxiety increased as adverse childhood experiences increased, and CAQ scores were found to be higher in primary school graduates, those with low income, those whose pregnancy was not planned, and those who used alcohol during pregnancy. These findings are consistent with evidence, indicating that early-life adversity may shape not only psychological attitudes toward childbirth but also women’s subjective experiences during labour and postpartum functioning. Do et al. (2022) found that women with a history of childhood abuse and prenatal intimate partner violence were significantly more likely to report negative childbirth experiences and reduced breastfeeding outcomes [35].

Pregnancy is the best time to identify current health risks in women, assess risky health behaviours, and provide interventions to prevent future health problems. Our findings showed that the mean score of the HPLP-II among pregnant women was 138.64 ± 23.71, which indicates a moderately good level of health-promoting behaviours. Among the HPLP-II subdimensions, the highest mean scores were observed in spiritual growth and interpersonal relations, followed by nutrition, health responsibility, stress management, and physical activity, which had the lowest mean score. Similar findings were reported by Rafat et al. (2024) and Pazandeh et al. (2024); however, the consistently low score in the physical activity dimension is noteworthy [37,38]. Therefore, healthcare professionals providing prenatal care should encourage pregnant women to engage in physical activity. Also, the HPLP-II score of pregnant women was found to be significantly higher in those who had a university degree or higher education, were employed, had a good income perception, had a planned last pregnancy, and did not smoke or drink alcohol during pregnancy. Similarly, Maulina et al. (2025) conducted a study in Indonesia and reported that pregnant women with higher education, employment, and average or above-average income demonstrated significantly better health-promoting behaviours [39]. Rafat et al. (2024) found that health literacy, education, employment, and participation in childbirth preparation classes were significant predictors of health-promoting lifestyle behaviours among pregnant women [37]. These findings underscore the importance of supporting women’s education and employment as key strategies to enhance health-promoting behaviours during pregnancy.

As the level of childhood adverse experiences increased, the HPLP-II scale score decreased. ACEs are known to cause an increase in risky health behaviours [20,33]. Currie et al. suggest that the likelihood of a woman consuming alcohol or engaging in binge drinking at least once during pregnancy increases in a dose-dependent manner with the number of ACEs experienced [20]. In addition, ACEs pose a risk for having multiple sexual partners, having first sexual intercourse at an earlier age, adolescent pregnancy, unintended pregnancies and sexually transmitted infections [12,32], and lower rates of prenatal care [32]. Many health behaviours predisposing individuals to preventable diseases have been associated with negative childhood experiences, such as alcohol consumption, drug use, and smoking. Entering pregnancy with a high ACE score may explain negative emotions, increased childbirth anxiety, and increased unhealthy lifestyle behaviours. [35].

Future research should explore the effectiveness of such interventions through longitudinal or randomized controlled designs. In addition, the inclusion of potential moderating or mediating variables such as partner violence, comorbid mental health conditions, and social support networks may provide a more comprehensive understanding of the pathways through which ACEs influence maternal and perinatal health.

### Limitations

This study has several limitations that should be considered when interpreting the findings. First, the research was conducted in a single geographic region, which may limit the generalizability of the results to broader or culturally diverse populations. Second, the use of self-reported data to assess adverse childhood experiences and psychological constructs introduces potential recall and social desirability biases. These biases may have led to underreporting or distortion of sensitive experiences, particularly childhood adversity. Future research using objective assessments or multiple data sources could improve the validity of measurements.

## Conclusion

This study demonstrates that adverse childhood experiences (ACEs) are significant predictors of both childbirth attitudes and health-promoting lifestyle behaviours in pregnant women. Integrating ACE screening into preconception and prenatal care may help reduce mental health risks and adverse pregnancy outcomes. Future research should explore the intergenerational effects of maternal ACE exposure, particularly on child development. These findings support the life course perspective, highlighting how early-life adversities can shape health-related behaviours and psychological orientations in adulthood.

## References

[1] Çiçek E, Karagözoğlu Ş. Gebelik sürecinde roy’un adaptasyon modeli doğrultusunda uygulanan hemşirelik bakımı: olgu sunumu. Cumhuriyet Üniversitesi Sağlık Bilimleri Enstitüsü Dergisi. 2024;9(1):115–23. doi: 10.51754/cusbed.1377291

[2] Akkaş MB, Ege E. Primipar ve Multipar Gebelerin Sağlıklı Yaşam Biçimi Davranışlarının İncelenmesi: Karşılaştırmalı Bir Çalışma. HUSBFD. 2023;10(2):341–51. doi: 10.21020/husbfd.1082464

[3] Mihiret GT, Meselu BT, Wondmu KS, Getaneh T, Moges NA. Health-promoting lifestyle behaviors and their associated factors among pregnant women in Debre Markos, northwest Ethiopia: a cross-sectional study. Front Glob Womens Health. 2025;5:1468725. doi: 10.3389/fgwh.2024.1468725 39830937 PMC11739096

[4] Yılar Erkek Z, Öztürk Altınayak S. Gebelerde E-Sağlık Okuryazarlığı ile Sağlıklı Yaşam Davranışları Arasındaki İlişki. Ebelik ve Sağlık Bilimleri Dergisi. 2024;7(2):282–92. doi: 10.62425/esbder.1509148

[5] Fathnezhad-Kazemi A, Aslani A, Hajian S. Association between perceived social support and health-promoting lifestyle in pregnant women: a cross-sectional study. J Caring Sci. 2021;10(2):96–102. doi: 10.34172/jcs.2021.018 34222119 PMC8242291

[6] Hughes K, Hardcastle K, Bellis MA. 286 The impact of adverse childhood experiences on health: a systematic review and meta-analysis. In: Child Maltreatment. 2016. doi: 10.1136/injuryprev-2016-042156.286

[7] Walker SN, Hill-Polerecky D. Psychometric evaluation of the health-promoting lifestyle profile II. University of Nebraska Medical Center. 1996. 120–6.

[8] Racine NM, Madigan SL, Plamondon AR, McDonald SW, Tough SC. Differential Associations of Adverse Childhood Experience on Maternal Health. Am J Prev Med. 2018;54(3):368–75. doi: 10.1016/j.amepre.2017.10.028 29306559

[9] Lin L, Wang HH, Lu C, Chen W, Guo VY. Adverse Childhood Experiences and Subsequent Chronic Diseases Among Middle-aged or Older Adults in China and Associations With Demographic and Socioeconomic Characteristics. JAMA Netw Open. 2021;4(10):e2130143. doi: 10.1001/jamanetworkopen.2021.30143 34694390 PMC8546496

[10] Petruccelli K, Davis J, Berman T. Adverse childhood experiences and associated health outcomes: A systematic review and meta-analysis. Child Abuse & Neglect. 2019;97:104127. doi: 10.1016/j.chiabu.2019.104127 31454589

[11] Hunt TKA, Slack KS, Berger LM. Adverse childhood experiences and behavioral problems in middle childhood. Child Abuse Negl. 2017;67:391–402. doi: 10.1016/j.chiabu.2016.11.005 27884508 PMC5436949

[12] Young-Wolff KC, Wei J, Varnado N, Rios N, Staunton M, Watson C. Adverse Childhood Experiences and Pregnancy Intentions among Pregnant Women Seeking Prenatal Care. Womens Health Issues. 2021;31(2):100–6. doi: 10.1016/j.whi.2020.08.012 33032888 PMC8005410

[13] Sperlich M, Seng JS, Li Y, Taylor J, Bradbury-Jones C. Integrating Trauma-Informed Care Into Maternity Care Practice: Conceptual and Practical Issues. J Midwifery Womens Health. 2017;62(6):661–72. doi: 10.1111/jmwh.12674 29193613

[14] Porthan E, Lindberg M, Härkönen J, Scheinin NM, Karlsson L, Karlsson H, et al. Childhood trauma and fear of childbirth: findings from a birth cohort study. Arch Womens Ment Health. 2023;26(4):523–9. doi: 10.1007/s00737-023-01328-x 37243781 PMC10333423

[15] Li B, Liu T, Ma D, Sun J, Liu J. Association of fear of childbirth and postpartum depression with perceived partner response during pregnancy. BMC Pregnancy Childbirth. 2025;25(1):211. doi: 10.1186/s12884-025-07332-6 40011837 PMC11863454

[16] Abdelaziz EM, Alshammari AM, Elsharkawy NB, Oraby FA, Ramadan OME. Digital intervention for tokophobia: a randomized controlled trial of internet-based cognitive behavioral therapy on fear of childbirth and self-efficacy among Egyptian pregnant women. BMC Pregnancy Childbirth. 2025;25(1). doi: 10.1186/s12884-025-07341-5 PMC11877725 40033245

[17] Swedo EA, D’Angelo DV, Fasula AM, Clayton HB, Ports KA. Associations of Adverse Childhood Experiences With Pregnancy and Infant Health. Am J Prev Med. 2023;64(4):512–24. doi: 10.1016/j.amepre.2022.10.017 36697281 PMC10033436

[18] Özşahin Z. The effects of adverse childhood experiences on pregnancy-related anxiety and acceptance of motherhood role. Afr H Sci. 2020;20(3):1217–28. doi: 10.4314/ahs.v20i3.25 PMC7751528 33402968

[19] Tran N, Callaway L, Shen S, Biswas T, Scott JG, Boyle F, et al. Screening for adverse childhood experiences in antenatal care settings: A scoping review. Aust N Z J Obstet Gynaecol. 2022;62(5):626–34. doi: 10.1111/ajo.13585 35909247 PMC9796324

[20] Currie CL, Sanders JL, Swanepoel L-M, Davies CM. Maternal adverse childhood experiences are associated with binge drinking during pregnancy in a dose-dependent pattern: Findings from the All Our Families cohort. Child Abuse Negl. 2020;101:104348. doi: 10.1016/j.chiabu.2019.104348 31896532

[21] McKelvin G, Thomson G, Downe S. The childbirth experience: A systematic review of predictors and outcomes. Women Birth. 2021;34(5):407–16. doi: 10.1016/j.wombi.2020.09.021 33039281

[22] Halfon N, Hochstein M. Life course health development: an integrated framework for developing health, policy, and research. Milbank Q. 2002;80(3):433–79, iii. doi: 10.1111/1468-0009.00019 12233246 PMC2690118

[23] von Elm E, Altman DG, Egger M, Pocock SJ, Gøtzsche PC, Vandenbroucke JP, et al. The Strengthening the Reporting of Observational Studies in Epidemiology (STROBE) statement: guidelines for reporting of observational studies. Internist (Berl). 2008;49(6):688–93. doi: 10.1007/s00108-008-2138-4 18511988

[24] Felitti VJ, Anda RF, Nordenberg D, Williamson DF, Spitz AM, Edwards V, et al. Relationship of childhood abuse and household dysfunction to many of the leading causes of death in adults. The Adverse Childhood Experiences (ACE) Study. Am J Prev Med. 1998;14(4):245–58. doi: 10.1016/s0749-3797(98)00017-8 9635069

[25] Gervin DW, Holland KM, Ottley PG, Holmes GM, Niolon PH, Mercy JA. Centers for Disease Control and Prevention Investments in Adverse Childhood Experience Prevention Efforts. American Journal of Preventive Medicine. 2022;62(6):S1–5. doi: 10.1016/j.amepre.2021.11.014 PMC11219185 35597578

[26] Gunduz A, Yasar AB, Gundogmus I, Savran C, Konuk E. Çocukluk çağı olumsuz yaşantılar ölçeği türkçe formunun geçerlilik ve güvenilirlik çalışması. Anadolu Psikiyatri Dergisi. 2018;19(1):68–75.

[27] Walker SN, Sechrist KR, Pender NJ. The Health-Promoting Lifestyle Profile. Nursing Research. 1987;36(2):76???81. doi: 10.1097/00006199-198703000-00002 3644262

[28] Bahar Z, Beser A, Gordes N, Ersin F, Kıssal A. Sağlıklı yaşam biçimi davranışları ölçeği II’nin geçerlik ve güvenirlik çalışması. Cumhuriyet Üniversitesi Hemşirelik Yüksekokulu Dergisi. 2008;12(1):1–13.

[29] Lowe NK. Self-efficacy for labor and childbirth fears in nulliparous pregnant women. Journal of Psychosomatic Obstetrics & Gynecology. 2000;21(4):219–24. doi: 10.3109/01674820009085591 11191169

[30] Donmez S, Dag H, Celik N, Yeniel A, Kavlak O. Doğum tutum ölçeğinin geçerlilik ve güvenilirlik çalışması. Journal of Clinical Obstetrics & Gynecology. 2014;24(4):212–2018.

[31] Chang X, Jiang X, Mkandarwire T, Shen M. Associations between adverse childhood experiences and health outcomes in adults aged 18-59 years. PLoS One. 2019;14(2):e0211850. doi: 10.1371/journal.pone.0211850 30730980 PMC6366931

[32] Testa A, Jackson DB, Ganson KT, Nagata JM. Maternal adverse childhood experiences and pregnancy intentions. Ann Epidemiol. 2021;64:47–52. doi: 10.1016/j.annepidem.2021.09.011 34547446

[33] Sherin KM, Stillerman AJ, Chandrasekar L, Went NS, Niebuhr DW. Recommendations for Population-Based Applications of the Adverse Childhood Experiences Study: Position Statement by the American College of Preventive Medicine. AJPM Focus. 2022;1(2):100039. doi: 10.1016/j.focus.2022.100039 37791246 PMC10546534

[34] Foti TR, Watson C, Adams SR, Rios N, Staunton M, Wei J, et al. Associations between Adverse Childhood Experiences (ACEs) and Prenatal Mental Health and Substance Use. Int J Environ Res Public Health. 2023;20(13):6289. doi: 10.3390/ijerph20136289 37444136 PMC10341286

[35] Loxton D, Forder PM, Cavenagh D, Townsend N, Holliday E, Chojenta C, et al. The impact of adverse childhood experiences on the health and health behaviors of young Australian women. Child Abuse Negl. 2021;111:104771. doi: 10.1016/j.chiabu.2020.104771 33160649

[36] Nilsson C, Hessman E, Sjöblom H, Dencker A, Jangsten E, Mollberg M, et al. Definitions, measurements and prevalence of fear of childbirth: a systematic review. BMC Pregnancy Childbirth. 2018;18(1). doi: 10.1186/s12884-018-1659-7 PMC5766978 29329526

[37] Rafat N, Bakouei F, Adib-Rad H, Nikbakht H-A, Bakouei S. Predicting the Health-Promoting Lifestyle Profile of Pregnant Women Based on Their Health Literacy Levels: A Cross-Sectional Study. Nurs Open. 2025;12(1):e70136. doi: 10.1002/nop2.70136 39731456 PMC11681366

[38] Pazandeh F, Banihashem F, Mohseni S, Mohseni M, Firouzi H. Social determinants of health and health-promoting lifestyle of pregnant women in Hormozgan province, southern Iran. Journal of Preventive Medicine. 2024;10(4):412–25.

[39] Maulina R, Kuo S-C, Liu C-Y, Lu YY, Khuzaiyah S, Caparros-Gonzalez RA. Does attachment and prenatal depression affect maternal health-promoting lifestyle during pregnancy? A cross-sectional study. Clinical Epidemiology and Global Health. 2025;31:101904. doi: 10.1016/j.cegh.2024.101904

